# Sialic acid-binding lectin (leczyme) induces caspase-dependent apoptosis-mediated mitochondrial perturbation in Jurkat cells

**DOI:** 10.3892/ijo.2013.2092

**Published:** 2013-09-05

**Authors:** TAKEO TATSUTA, MASAHIRO HOSONO, SHIGEKI SUGAWARA, YUKIKO KARIYA, YUKIKO OGAWA, SENITIROH HAKOMORI, KAZUO NITTA

**Affiliations:** 1 Division of Cell Recognition Study, Institute of Molecular Biomembrane and Glycobiology, Tohoku Pharmaceutical University, Aoba-ku, Sendai 981-8558;; 2 Fukushima Medical University, Fukushima 960-1295;; 3 Divisions of Functional Morphology and Microbiology, Department of Pharmacy, Faculty of Pharmaceutical Science, Nagasaki International University, Sasebo, Nagasaki 859-3298, Japan;; 4 Division of Biomembrane Research, Pacific Northwest Research Institute, Seattle, WA 98122, USA

**Keywords:** lectin, ribonuclease, antitumor effect, leczyme, *Rana catesbeiana*, caspase pathway, mitochondria perturbation

## Abstract

Sialic acid binding lectin (SBL) isolated from *Rana catesbeiana* oocytes is a multifunctional protein which has lectin activity, ribonuclease activity and antitumor activity. However, the mechanism of antitumor effects of SBL is unclear to date and the validity for human leukemia cells has not been fully studied. We report here that SBL shows cytotoxicity for some human leukemia cell lines including multidrug-resistant (MDR) cells. The precise mechanisms of SBL-induced apoptotic signals were analyzed by combinational usage of specific caspase inhibitors and the mitochondrial membrane depolarization detector JC-1. It was demonstrated that SBL causes mitochondrial perturbation and the apoptotic signal is amplified by caspases and cell death is executed in a caspase-dependent manner. The efficacy of this combinational usage was shown for the first time, to distinguish the apoptotic pathway in detail. SBL selectively kills tumor cells, is able to exhibit cytotoxicity regardless of P-glycoprotein expression and has potential as an alternative to conventional DNA-damaging anticancer drugs.

## Introduction

Historically, replicative DNA was the main target of anti-cancer agents for many years, but because of the selectivity or occurrence of resistance to the drugs, agents which have new strategy such as molecular targeted therapy show promise for the treatment of cancer. Efforts to improve cancer therapy have focused on the development of more selective, biological mechanism-based agents that can overcome tumor resistance, as well as minimize toxic effects to normal cells (1).

Ribonucleases are enzymes which catalyze the degradation of RNA. RNases display various biological roles, including nutritional function, remobilization of phosphate, senescence, self-incompatibility, defensin-like activity and the conspicuous function in RNA metabolism (2,3). Some members of RNases are reported to exhibit angiogenic, neurotoxic, antitumor, or immunosuppressive activities (4). Bovine pancreatic ribonuclease A (RNase A), EC 3.1.27.5 (5), was the first RNase tested for a possible anticancer activity *in vitro* (6–8) and *in vivo* (9–12). While RNase A needed high amounts to observe the anticancer activity, more effective RNases have been reported in recent years. The proposed mechanism of ribonuclease-induced cytotoxicity is: i) cell surface binding and internalization, ii) translocation to the cytosol, iii) evasion of the cytosolic ribonuclease inhibitor protein (RI) and iv) degradation of cellular RNA. Differences in the efficiency of any of these steps could affect the cell susceptibility (13). One promising RNase for cancer therapeutic drug is onconase, a ribonuclease isolated from *Rana pipiens* oocytes. Onconase, manifests cytotoxic and cytostatic effects (14), presents synergism with several kinds of anti-cancer drugs (15–22) and at present is in phase II/III clinical trials as an anticancer drug (1,23). Onconase has demonstrated some advantages for potential clinical applications, including: a) evading human RNase inhibitors in cytosol, b) inhibitory activity against broad types of human tumors, c) without any untoward immune response and exerting only weak and reversible renal toxicity (24). The phase III clinical trial of onconase has prompted the genetic engineering of known RNases as well as a search for new medicinal RNases (3,12,24,25).

Sialic acid binding lectin (SBL) isolated from *R. catesbeiana* oocytes was found as a lectin, because SBL agglutinates various kinds of tumor cells and the agglutination was inhibited by sialoglycoprotein or ganglioside (26–28). Agglutination induced by SBL was observed in tumor cells, but not in normal red blood cells or fibroblasts (28). Amino acid sequence of SBL shows that it has homology to the member of RNase A superfamily and it has been revealed that SBL practically has pyrimidine base-specific ribonuclease activity (29–32). The antitumor effect of SBL was reported using P388 and L1210 murine leukemia cells *in vitro* and sarcoma 180, Ehrlich and Mep 2 ascites cells *in vivo* (33–35). RC-RNase isolated from *R. catesbeiana* is identical to SBL (36,37). It was also reported that RC-RNase seems to harbor a more specific anticancer activity compared with onconase (38).

However, the mechanism of antitumor effect of SBL is unclear and the validity for human leukemia cells has not been fully studied. We studied the antitumor effect of SBL using some human leukemia cell lines. We found that SBL shows cytotoxicity to some cell lines, including multiple drug resistant (MDR) cells. The mechanism of SBL-induced cytotoxicity is analyzed in detail by combinational usage of specific caspase inhibitors and mitochondrial membrane depolarization detector JC-1 and we clearly show that cytotoxicity is induced through caspase-dependent apoptosis in which mitochondrial perturbation occurs as upstream events. It is extrapolated that the novel mechanistic apoptosis inducing activity toward various human leukemia cells regardless of P-glycoprotein (P-gp) expression indicating that SBL is a new candidate as an alternative to conventional DNA-damaging anticancer drugs.

## Materials and methods

### Materials

SBL was isolated in sequential chromatography on Sephadex G-75, DEAE-cellulose, hydroxyapatite and SP-Sepharose as described previously (28). Etoposide (ETO), doxorubicin (DOX) and anti-β-actin antibody were purchased from Sigma-Aldrich (Tokyo, Japan). Tumor necrosis factor-related apoptosis inducing ligand (TRAIL) was purchased from R&D Systems (Minneapolis, MN, USA). Caspase inhibitors (zVAD-fmk, zIETD-fmk, zLEHD-fmk) and anti-caspase-9 antibody were purchased from Medical & Biological Laboratories Co., Ltd. (MBL, Nagoya, Japan). Anti-caspase-8 antibody, anti-caspase-3 antibody and anti-Bid antibody were purchased from Cell Signaling Technology (Beverly, MA, USA). Anti-cytochrome *c* antibody was purchased from Becton-Dickinson (Franklin Lakes, NJ, USA). Horseradish peroxidase (HRP)-conjugated anti-mouse IgG actibody and HRP-conjugated anti-rabbit IgG andibody was purchased from Zymed (South San Francisco, CA, USA) and Cedarlane Lab. Ltd. (Hornby, Ontario, Canada), respectively.

### Cell culture

Human leukemia Jurkat T-cells, erythroleukemia K562 cells, Adriamycin-resistant and P-gp-overexpressing K562 cells (K562/ADR), Burkitt’s lymphoma Raji cells and promyelocytic leukemia U937 cells were obtained from the Cell Resource Center of the Biomedical Research, Institute of Development, Ageing and Cancer, Tohoku University (Sendai, Japan). Cells were routinely kept in RPMI-1640 medium (Nissui Pharmaceutical Co. Ltd., Tokyo, Japan) supplemented with 10% fetal calf serum (FCS), penicillin (100 U/ml) and streptomycin (100 *μ*g/ml) at 37°C in a 95% air and 5% CO_2_ atmosphere.

### RNA extraction and analysis

The cells were treated with SBL (2 *μ*M) for indicated time. Total RNA of the cells was extracted with TRIzol reagent (Invitrogen, Carlsbad, CA, USA) according to the manufacturer’s instructions. RNA (1 *μ*g) was electrophoresed on 2% agarose gel containing formaldehyde (18%). The gels were visualized by ethidium bromide staining.

### Measurement of cell viability

To determine the cytotoxicity, WST-8 assays (39), were done in accordance with the manufacturer’s instructions. Briefly, the cells (2×10^4^ cells/well) were plated into 96-well plates. Various concentration of reagents were added in triplicate to the cultures and incubated for indicated times before adding the WST-8 solution. The absorbance of the resulting product was measured 4 h later at a wavelength of 450 nm with back ground subtraction at 650 nm. The IC_50_ which shows the compound concentration required for 50% inhibition of the cell growth was calculated employing GraphPad Prism 3.0 software. Cell viability was determined by trypan blue dye exclusion assay. The cells (2×10^5^ cells/ml) were cultured in 100 *μ*l in 96-well plates. After treatment with SBL, the cells were stained with 0.25% trypan blue and both viable and non-viable cells were counted.

### Observation of nuclear morphology

The cells (2×10^5^ cells/ ml) were cultured in 5 ml in 6-well plates. After treatment with SBL, the cells were collected by centrifugation and washed with PBS. Then the cells were fixed with 1% paraformaldehyde (100 *μ*l) for 15 min at 4°C, and stained with Hoechst 33258 (50 *μ*l, 1 mg/ml) for 15 min at 4°C. After three washes with PBS, the cells were mounted on slide glass using Prolong gold antifade reagent (Molecular Probes). The fluorescence was visualized with a fluorescence microscope, Zeiss Axioscope 2 (Carl Zeiss, Jena GmbH, Jena, Germany).

### Detection of DNA fragmentation

The cells (2×10^5^ cells/ml) were cultured in 100 *μ*l in 96-well plates. After treatment with SBL, the cells were collected by centrifugation, washed with PBS, then lysed with cell lysis buffer [50 mM Tris-HCl (pH 6.8), 10 mM EDTA, 0.5% w/v sodium-N-lauroylsarcosinate]. The samples were incubated for 30 min with RNase A (final concentration, 500 *μ*g/ml) at 50°C, before being digested for 30 min with proteinase K (final concentration, 500 *μ*g/ml) at 50°C. Then the samples were electrophoresed on 1.8% agarose gel, DNA bands were visualized by ethidium bromide staining.

### Flow cytometric analysis of Annexin V binding and propidium iodide (PI) incorporation

Annexin V binding and PI incorporation were detected with a MEBCYTO apoptosis kit (MBL) according to the manufacturer’s instructions. The cells (2×10^5^ cells/ml) were cultured in 1 ml in 24-well plates. Fluorescence intensity of fluorescein isothiocyanate (FITC)-Annexin V and PI was determined using a FACSCalibur flow cytometer (Becton-Dickinson).

### Detection of caspase activity

Caspase activity was measured with caspase colorimetric protease assay kit (MBL) in accordance with the manufacturer’s instructions. After treatment with SBL (2 *μ*M) for indicated time, cells were lysed with cell lysis buffer and incubated for 10 min at 4°C. Then samples were centrifuged at 12,400 rpm and supernatant was collected. Samples (50 *μ*l, 1 *μ*g/*μ*l) were mixed with equal amount of 2X reaction buffer and substrates (DEVD-pNA for caspase-3, LEHD-pNA for caspase-9, IETD-pNA for caspase-8) were added at final concentration 200 *μ*M. After incubation for 2 h at 37°C, the absorbance of the resulting product was measured at a wavelength of 405 nm.

### Treatment of caspase inhibitors

The role of caspase activation in the process was studied by the addition of zVAD-fmk (pancaspase inhibitor), zIETD-fmk (caspase-8 specific inhibitor) and zLEHD-fmk (caspase-9 specific inhibitor). Each caspase inhibitor (50 *μ*M) was added to culture medium 30 min before the addition of reagents.

### Western blotting

Whole cell lysate was prepared by lysing the cells with extraction buffer [150 mM NaCl, 1% Triton X-100, 10 mM Tris-HCl (pH 7.5), 5 mM EDTA (pH 8.0), 1 mM phenylmethylsulfonyl fluoride (PMSF), 1 tablet/10 ml protease inhibitor cocktail (Roche Applied Science, Indianapolis, IN, USA)]. Lysates of organelle (mitochondria) fraction or cytosol fraction were prepared by ProteoExtract Subcellular Proteome Extraction Kit (Merck Millipore, Billerica, MA, USA). Soluble proteins were collected and concentrations were measured by DC protein assay kit (Bio-Rad, Richmond, CA, USA) in accordance with instructions. Proteins were separated by SDS-PAGE and transferred to polyvinylidene difluoride (PVDF) membrane (GE Healthcare, Little Chalfont, UK). The membrane was blocked by 5% fat-free skim milk for 1 h. After the membrane was washed with TBST [20 mM Tris-HCl (pH 7.6), 137 mM NaCl, 0.05% Tween-20], primary and secondary antibodies were added to the membrane, respectively. The proteins on membrane were detected using ECL western blotting detection regents (GE Healthcare).

### Detection of mitochondrial membrane potential (MMP) reduction

MMP was assessed using a fluorescent probe 5,50,6,60-tetrachloro-1,10,3,30-tetraethyl-benzamidazolocarbocyanin iodide (JC-1, AnaSpec, Fremont, CA, USA). Red emission from the dye is attributed to a potential-dependent aggregation of JC-1 in the mitochondria. Green fluorescence reflects the monomeric form of JC-1, appearing in the cytoplasm after mitochondrial membrane depolarization. Cells were cultured in condition of each experiment and then incubated with JC-1 (2 *μ*M) dye diluted in culture medium at 37°C for 15 min. The cells were washed three times with PBS and analyzed immediately using FACSCalibur (Becton-Dickinson).

### Statistical analysis

Results were collected from three independent experiments, each performed in triplicate and data are expressed as mean ± SD. Statistical analysis was performed using GraphPad Prism 3.0 and comparisons were made using one-way or two-way analysis of variance (ANOVA), followed by Bonferroni’s *post hoc* tests.

## Results

### SBL shows cytotoxicity to some human leukemia cell lines including MDR cells

We have previously shown that SBL has antitumor activity against mouse leukemia cells *in vitro* and *in vivo* (33,34,40). In this study, antitumor activity of SBL for human leukemia cell line and the signaling mechanism was evaluated. To determine the effect of SBL on cell viability of some human leukemia cell lines, WST assay was performed. As shown in Table I, SBL shows cytotoxic effect for all cell lines tested at low concentration (0.15–1.39 *μ*M) and the lowest IC_50_ value was observed in Jurkat cells (0.15 *μ*M). The cytotoxic effect of SBL was observed regardless of the P-gp expression level, while ETO and DOX which are used clinically for leukemia as DNA damaging agent did not cause cytotoxic effect on P-gp-overexpressing K562 cells.

### SBL degrades cellular RNA and inhibits cell proliferation of Jurkat cells

**Figure 1.**
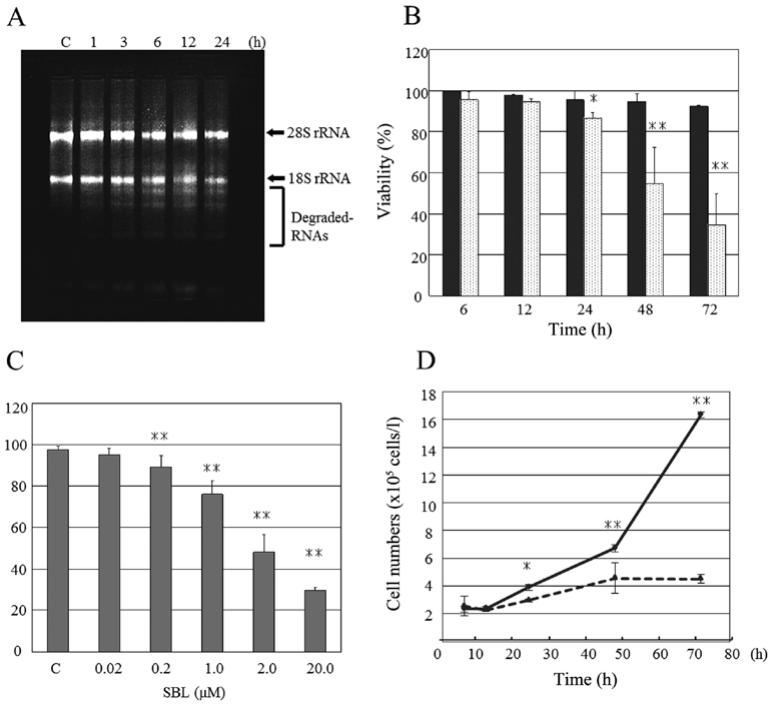
SBL degrades cellular RNA and inhibits cell proliferation of Jurkat cells. (A) Cells were treated with SBL (2 *μ*M) for indicated time and RNA was extracted. Then, electrophoresis was performed on 1.5% agarose gel. C, control. Degraded RNAs were seen under 18S rRNA. (B) Cells were treated with (doted column) or without (filled column) SBL (2 *μ*M) for indicated time. Cell viability was evaluated by trypan blue dye exclusion assay. (C) Cells were treated with SBL at various concentrations for 48 h. Cell viability was evaluated by the method described above. C, control. (D) Cells were treated with (dotted line) or without (solid line) SBL (2 *μ*M) for indicated time and total cell number were counted. Each value represents the mean ± SD of three independent experiments. ^*^p<0.05 and ^**^p<0.01 vs. control.

Because SBL has pyrimidine base-specific ribonuclease activity (31,32), we analyzed whether the cellular RNA is degraded by SBL or not. Total RNA extracted from SBL-treated Jurkat cells was analyzed by agarose gel electrophoresis and partial RNA degradation was found from 3-h treatment, then increased time-dependently (Fig. 1A). Next, the effects of SBL on Jurkat cell proliferation were analyzed in detail. Trypan blue dye exclusion assay showed that SBL (2 *μ*M) exhibited cytotoxicity toward Jurkat cells from 24 h time-dependently and that SBL (0.2 *μ*M or above) exhibited cytotoxicity in 48-h treatment concentration-dependently (Fig. 1B and C). Furthermore, significant inhibition of Jurkat cell proliferation was observed from 24-h treatment (Fig. 1D).

### SBL induces apoptosis in Jurkat cells

**Figure 2.**
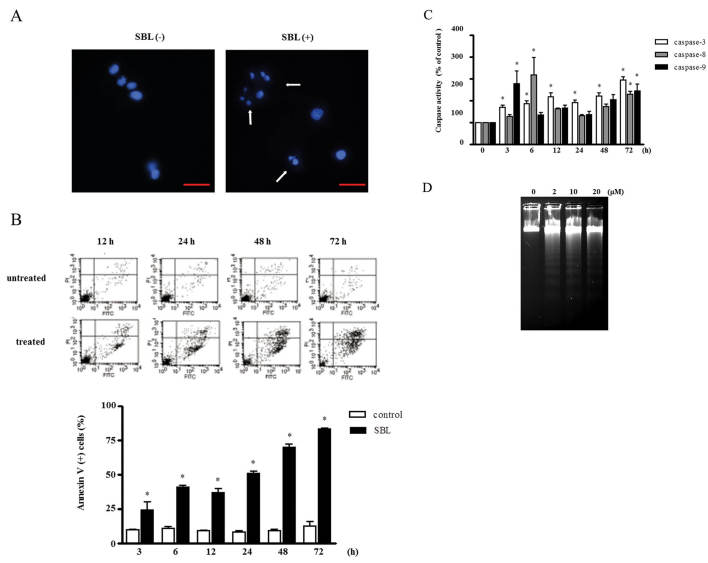
SBL induces apoptosis in Jurkat cells. (A) Morphological changes of nuclei in SBL-treated Jurkat cells. Cells were treated with SBL (2 *μ*M) for 48 h and stained with Hoechst 33258. Nuclei were observed using a fluorescent microscope. SBL-untreated (left panel) and -treated (right panel) cells. Arrows indicate apoptotic nuclei. Magnification, ×40 (scale, 10 *μ*m). (B) Time-dependent changes of FITC-Annexin V binding and PI incorporation in SBL-treated Jurkat cells. Cells were treated with SBL (2 *μ*M) for indicated time. Then, analysis of Annexin V-bound vs. PI incorporated cells was performed by FACSCalibur. In the lower panel, the percentage of Annexin V-positive cells is presented. Each value is the mean ± SE of three independent experiments. ^*^p<0.05 vs. untreated cells. (C) Caspase activity of SBL-treated Jurkat cells. Cells were treated with SBL (2 *μ*M) for indicated time. Then, the activity of each caspase was measured using fluorometric assay kit. (D) DNA fragmentation in SBL-treated Jurkat cells. Cells were treated with SBL at indicated concentrations for 48 h and DNA was prepared from the cells. DNA fragmentation was analyzed by agarose gel electrophoresis and stained with ethidium bromide.

To study the mechanism involved in the cytotoxicity of SBL, first we investigated the morphological changes in SBL-treated Jurkat cells using Hoechst 33258. Exposure of SBL resulted in typical apoptotic morphological alterations, such as karyorrhexis, nuclear condensation and nuclear fragmentation (Fig. 2A). We further observed the apoptotic biological changes in SBL-treated Jurkat cells. Annexin-V binding which is attributed to externalization of phosphatidylserine (PS) was observed from 3-h treatment of SBL (Fig. 2B). Simultaneously, the activation of initiator caspase (−8 and −9) and effector caspase (−3) was observed from 3 or 6 h (Fig. 2C). Similarly, DNA fragmentation was observed in a dose-dependent manner (Fig. 2D). These data indicate that SBL induces apoptosis in Jurkat cells.

### SBL-induced apoptosis is dependent on caspases and caspase-9 is activated more strongly than caspase-8

**Figure 3.**
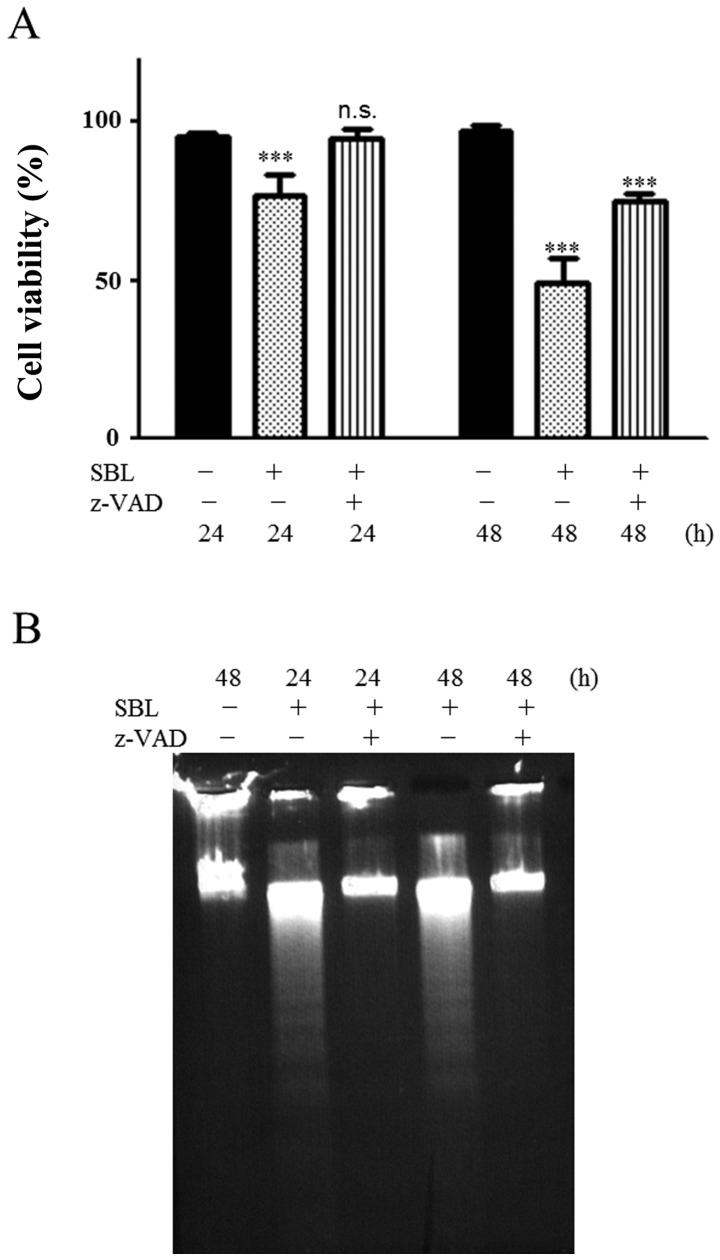
SBL-induced apoptosis is dependent on caspases. (A) Effect of z-VAD on SBL-induced cell death. Cells were treated with (striped column) or without (dotted column) z-VAD-fmk (50 *μ*M) for 30 min and subsequently treated with SBL (2 *μ*M) for indicated time. Cell viability was evaluated by trypan blue dye exclusion assay. Control (black column). Bars, mean ± SD. ^***^p<0.001. (B) Effect of z-VAD on DNA fragmentation in SBL-treated cells. Cells were treated with or without z-VAD-fmk (50 *μ*M) for 30 min and subsequently treated with SBL (2 *μ*M) for indicated time. Then, DNA was prepared from the cells. DNA fragmentation was analyzed by agarose gel electrophoresis and stained with ethidium bromide.

**Figure 4.**
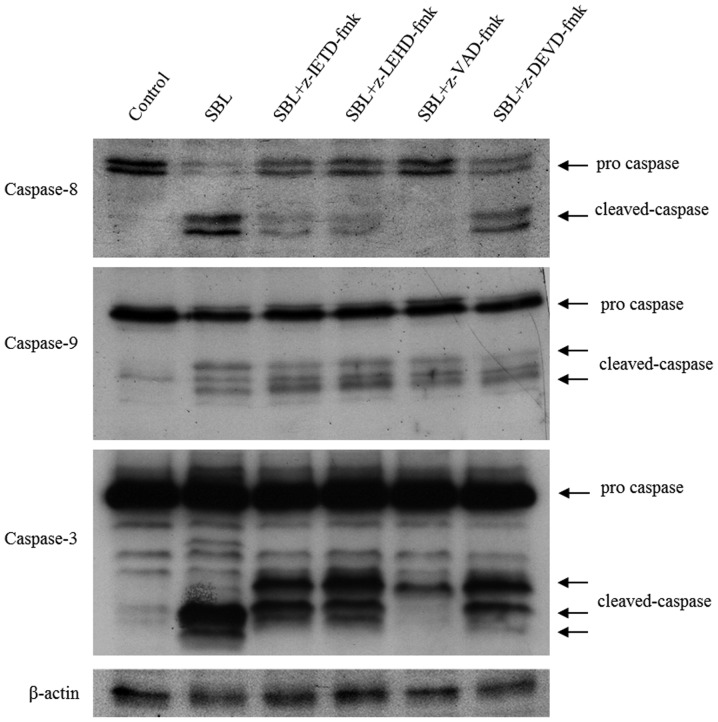
Effect of caspase inhibitors on SBL-induced caspase activation in Jurkat cells. Cells were treated with or without caspase inhibitor (50 *μ*M) for 30 min and subsequently treated with SBL (2 *μ*M) for 12 h. Then, whole cell lysate was separated by SDS-PAGE in 15% gel and subjected to western blot analysis using specific antibodies. β-actin was probed to demonstrate equal loading.

To analyze the detail of SBL-induced caspase activation, we performed experiments using caspase inhibitors. Pretreatment of z-VAD inhibited SBL-induced cell death (Fig. 3A) and completely blocked SBL-induced DNA fragmentation (Fig. 3B). Next, we analyzed activation pattern of caspase-8, -9 and -3 under the presence of specific inhibitors for each of the caspases (Fig. 4). Pretreatment of caspase-9 inhibitor, z-LEHD, inhibited activation of caspase-8. On the other hand, pretreatment of caspase-8 inhibitor, z-IETD, could not inhibit caspase-9 activation and the activation of caspase-9 was not affected even by the treatment of z-VAD or z-LEHD. Pretreatment of caspase-3 inhibitor, z-DEVD did not have an effect on activation of caspase-8 or -9. These data indicate that SBL induces caspase-dependent apoptosis and caspase-9 is activated strongly in SBL-induced apoptosis.

### SBL induces activation of p38 and JNK, but not ERK

**Figure 5.**
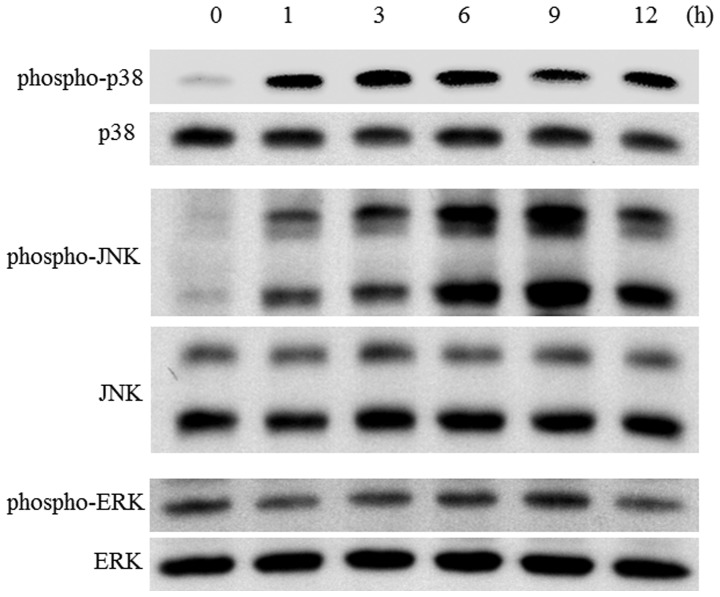
SBL induces activation of p38 and JNK but not activation of ERK. Cells were treated with SBL (2 *μ*M) for indicated time and phosphorylation status of p38, JNK and ERK were determined by western blot analysis. Expression of total p38, JNK and ERK was also determined to confirm equal amount of protein loading in each lane.

We monitored the activation of mitogen-activated protein kinases (MAPKs), extracellular signal-regulated kinases (ERKs), c-jun N-terminal kinase (JNKs)/stress-activated protein kinases and p38 in SBL-treated cells. Fig. 5 shows p38 kinase was activated as early as 1 h and sustained to 12 h. Furthermore, activation of JNK1/2 was observed from 1-h treatment and maximal at 6–9 h, whereas, activation of ERK was not observed in this condition. These results suggest that p38 and JNK may be involved in SBL-induced apoptotic signaling.

### Mitochondrial perturbation occurs before activation of caspases

**Figure 6.**
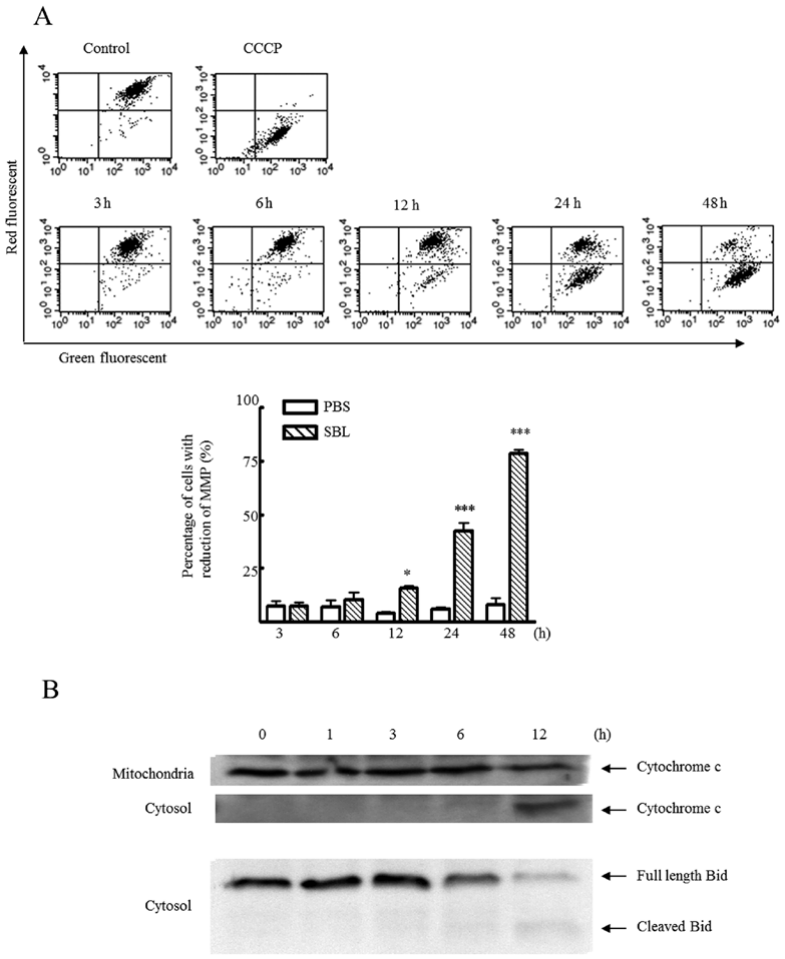
SBL induces mitochondrial perturbation in Jurkat cells. (A) Effect of SBL on MMP. Cells were treated with or without SBL (2 *μ*M) for indicated time. Then, the cells were stained with JC-1 (mitochondria selective dye) and analyzed using FACSCalibur. In lower panel, the percentage of cells with reduced MMP (lower right quadrant of each dot plot) was represented. Each value represents the mean ± SD of three independent experiments. (B) Release of cytochrome *c* from mitochondria to cytosol and Bid cleavage in SBL-treated cells. Cells were treated with SBL (2 *μ*M) for indicated time. Then, organelle and cytosol fractions were separated by SDS-PAGE in 15% gel and subjected to western blot analysis using anti-cytochrome *c* antibody and anti-Bid antibody.

Because caspase-9 is known as initiator caspase in apoptosis through mitochondria pathway, we analyzed the mitochondrial perturbation in SBL-induced apoptosis. During apoptosis, loss of mitochondrial membrane potential (MMP) is observed. We detected mitochondrial membrane depolarization using JC-1 fluorescent dye in SBL-treated Jurkat cells and found that SBL caused mitochondrial membrane depolarization in a time-dependent manner (Fig. 6A). At the same time, release of cytochrome *c* from mitochondria to cytosol was also observed (Fig. 6B) and the cleavage of Bid, which causes an efflux of cytochrome *c* from the mitochondria, was observed from 6 h. These results show occurrence of mitochondrial perturbation in SBL-treated Jurkat cells.

**Figure 7.**
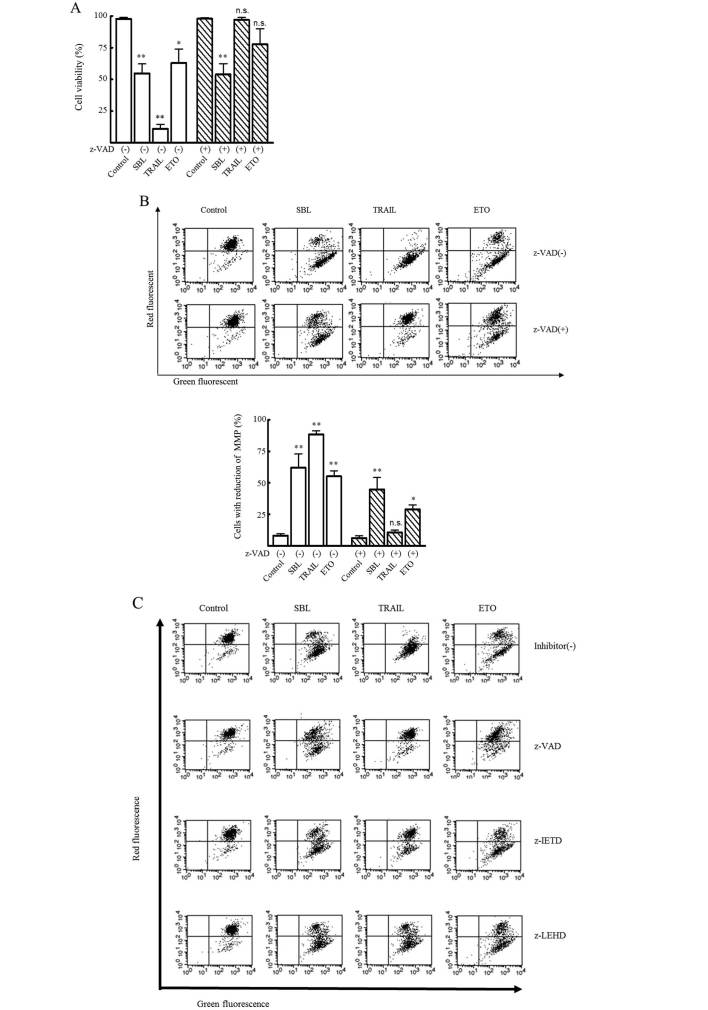
Mitochondrial perturbation occurs in advance of caspase activation. (A) Effect of z-VAD on viability of SBL-, TRAIL- or ETO-treated cells. Cell viability was evaluated by trypan blue dye exclusion assay. Cells were treated with or without z-VAD-fmk (50 *μ*M) for 30 min and subsequently treated with SBL (2 *μ*M), TRAIL (5 ng/ml) and ETO (200 *μ*M) for 48 h. (B) Effect of z-VAD-fmk on SBL-, TRAIL-, or ETO-induced loss of MMP. MMP were measured as described in Fig. 6. In the lower panel, the percentage of cells with reduced MMP is presented. Each value represents the mean ± SD of three independent experiments. (C) Effect of z-IETD or z-LEHD on loss of SBL-induced mitochondrial membrane potential. Cells were treated with or without each caspase inhibitor (50 *μ*M) for 30 min and subsequently treated with SBL (2 *μ*M), TRAIL (5 ng/ml) and ETO (200 *μ*M), for 48 h. Then, MMP was measured as described above.

To study the importance of mitochondrial perturbation in SBL-induced apoptosis, we analyzed cell viability and the mitochondrial depolarization under the presence of caspase inhibitors comparing with TRAIL and ETO known as inducer of apoptosis through death receptor pathway and mitochondrial pathway, respectively. At 48-h treatment with each of the reagents, pretreatment of z-VAD completely inhibited TRAIL-induced cytotoxicity, but did not or only partially inhibited SBL- or ETO-induced cytotoxicity (Fig. 7A). Similarly, mitochondrial depolarization caused by TRAIL was completely inhibited by z-VAD, while SBL or ETO-induced mitochondrial depolarization was not affected by z-VAD (Fig. 7B). Furthermore, z-IETD inhibited TRAIL-induced mitochondrial depolarization to similar extent with z-VAD, whereas, z-LEHD was less effective. On SBL- or ETO-induced mitochondrial depolarization, neither IETD nor LEHD showed inhibitory effect like z-VAD (Fig. 7C). These results indicate that SBL-induced mitochondrial perturbation is not dependent on caspase activation. Thus, SBL invokes mitochondrial perturbation first and this process is followed by caspase activation and the amplification of death signal executes apoptotic cell death.

## Discussion

**Figure 8.**
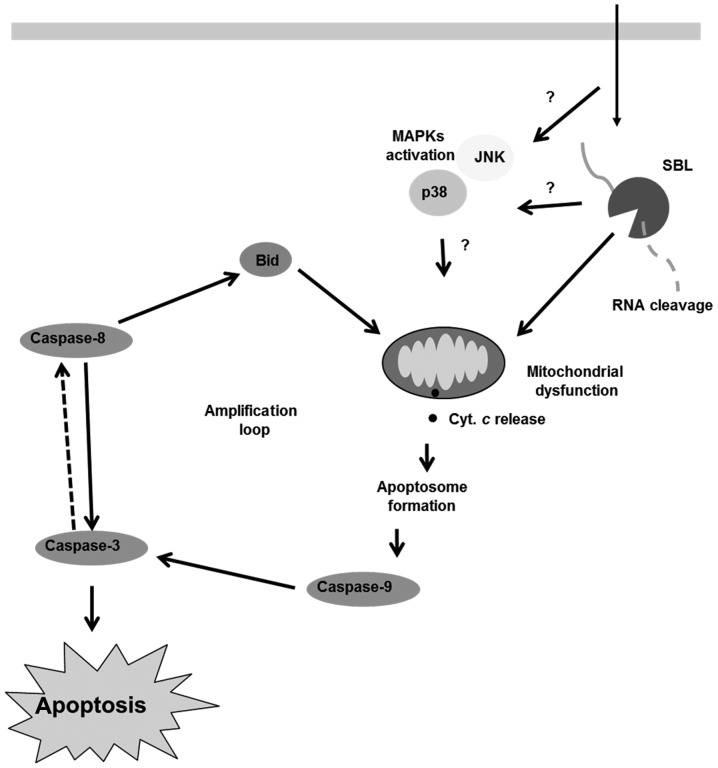
Proposed model for apoptotic mechanism induced by SBL in Jurkat cells. SBL binds to cell surface and internalizes into tumor cells. SBL degrades cellular RNA and this ribotoxic stress triggers mitochondrial perturbation. Then, apoptotic signal is amplified by caspase activation leading to cell death.

SBL is a multifunctional protein which has lectin activity, ribonuclease activity and antitumor activity. The proposed mechanism of SBL-induced cell death is shown in Fig. 8. SBL binds to cell surface, internalizes into tumor cells and degrades cellular RNA and this ribotoxic stress triggers mitochondrial perturbation. The activation of p38 and JNK may be involved in the above process. Then, apoptotic signal is amplified by activation of caspase and leads to cell death.

SBL selectively agglutinates tumor cells, but not erythrocytes or fibroblasts (28). SBL shows cytotoxity to various tumor cells, but not to human primary WI-38 lung fibroblasts, normal mesothelial Met-5A cells (data not shown), human primary HFW fibroblasts, immortalized murine NIH- 3T3/3 cells (40), human primary HS-68 foreskin fibroblasts (37) or hamster kidney BHK-21 cells (41). It seems that the selective effect of SBL on cancer cells is due to its selective binding to tumor cells, because sialidase treatment of cells abolished the tumor cell agglutination and also the antiproliferative effect induced by SBL (33).

In this study, we showed that SBL manifests cytotoxicity to some human leukemia cell lines including MDR cells, while conventional DNA-damaging agents, ETO and DOX which have been used clinically were not able to show cytotoxicity to MDR cells (Table I). The resistance of tumors occurs as a cross-resistance to a whole range of drugs with different structures and this phenomenon is called MDR. The cytotoxic drugs that are most frequently associated with MDR are hydrophobic and amphipathic natural products, such as the taxanes (paclitaxel and docetaxel), vinca alkaloids (vinorelbine, vincristine and vinblastine), anthracyclines (DOX, daunorubicin and epirubicin), epipodophyllotoxins (ETO and teniposide), antimetabolites (methorexate, fluorouracil, cytosar, 5-azacytosine, 6-mercaptopurine and gemcitabine), topotecan, dactinomycin and mitomycin *c* (42–46). Overexpression of ATP-binding cassette (ABC) transporters such as P-gp is known to be responsible for MDR (46). Cytotoxic RNase, PE5 (a variant of human pancreatic ribonuclease carrying a nuclear localization signal) reduced the expression level of the P-gp in MDR cell lines (47). It is believed that SBL displays novel mechanistic and tumor-selective cytotoxic effects regardless of P-gp expression and SBL is favorable as a new candidate anticancer drug.

Apoptosis, also known as programmed cell death, plays a critical role in various biological phenomena, such as development, immunity and also cell death induced by chemotherapeutic drugs (48). Apoptosis may occur through death receptor-dependent (extrinsic) or independent (intrinsic or mitochondrial) pathways and the pathways finally activate the effector caspase-3, which leads to finally execution of apoptosis (49). During the execution phase of apoptosis, typical apoptotic changes such as chromatin condensation, nuclear collapse, internucleosomal DNA fragmentation are observed. A variety of studies have demonstrated that in cancer therapy, induction of apoptosis is a frequent outcome of effective therapy. In this study, we showed SBL-treated Jurkat cells present typical apoptotic morphological alterations, such as karyorrhexis, nuclear condensation and fragmentation (Fig. 2A) and apoptotic biological changes such as PS externalization, activation of caspases, and DNA fragmentation (Fig. 2B–D). This SBL-induced DNA fragmentation was completely blocked by z-VAD indicating that the cytotoxicity of SBL is induced through caspase-dependent apoptosis.

It has been reported that some chemotherapeutics and natural toxins which induce ribotoxic stress response activates MAPK (50,51). Regarding ribotoxic stress, He *et al* demonstrated ribotoxins, such as deoxynivalenol (DON), anisomycin, satratoxin G (SG) and ricin activate p38, JNKs and ERK in RAW264 mouse macrophage cell line (52). It was reported that activation of JNK is important for cytotoxicity of onconase using *jnk1^−/−^ jnk2^−/−^* mouse embryo fibroblast (MEF) cells (53). Fang *et al* reported that RNase MC2 induces phosphorylation of p38, JNK and ERK in MCF-7 cells (3) and this RNase-mediated apoptotic signaling is contributed by dual phosphorylation of ERK and JNK in Hep G2 cells (54). Although it has been implicated largely that activation of p38 and JNK are proapoptotic (50,55) and that phosphorylation of ERK is linked with both antitumor activity (56) and tumor progression (57), some complicated results have been reported. Costro *et al* reported that PE5 kills adriamycin-resitant MCF-7 (MCF-7/ADR) cells through apoptosis associated with the inactivation of JNK, while onconase did not change the phosphorylation level of JNK in the cells (25). We showed that SBL is capable of inducing activation of p38 and JNK, but not ERK. The activation of p38 and JNK were observed as early as 1-h treatment with SBL suggesting that p38 and JNK may be activated upstream of mitochondrial perturbation. Although we tested the effects of p38 inhibitor (SP600125) and JNK inhibitor (SB203580), they did not affect the cytotoxicity induced by SBL (data not shown). There are some possible explanations for this phenomenon: i.e., binding to cell surface or the internalization into cytosol is able to display RNase activity of SBL, or the first cleavage of RNA, which is a non-detectable amount by electrophoresis can activate p38 and JNK. In addition, the activation of p38 and JNK may not be related to SBL cytotoxicity, or the inhibition of their activation may induce alternative death signals. The contribution of p38- and JNK-activation in SBL-induced cytotoxicity remains to be elucidated.

Wolf and Green reported that caspase-3 is capable of eliciting cleavage and activation of caspase-8 (58). Activation of caspase-8 results in the cleavage of Bid to produce a truncated form of the protein. Truncated Bid translocates from the cytoplasm to the mitochondria, where it appears to interact with and antagonize the actions of anti-apoptotic members of the Bcl-2 family, thereby causing an efflux of cytochrome *c* from the mitochondria (59–63). This, in turn, can result in the activation of caspase-9. Therefore, caspase-8 could amplify apoptotic signals through the continued release of cytochrome *c* and subsequent activation of caspase-9 and -3 (64). Once activating signals of apoptotic caspase are induced, the amplification signal can activate caspase-8, -9 and -3. In addition, the determination of exact time course of the sequential events is limited by detection sensitivity of experiments. These facts disturb discrimination of which pathway is involved in the stimuli. We utilized a combination of specific caspase inhibitors and mitochondrial membrane depolarization detector JC-1 to distinguish the SBL-induced signaling pathway comparatively with TRAIL and ETO. It was clearly shown that SBL-induced mitochondrial depolarization was not diminished by z-VAD, while TRAIL-induced mitochondrial depolarization was completely inhibited by z-VAD (Fig. 7A and B). These results indicate that cytotoxicity of SBL is induced through caspase-dependent apoptosis in which mitochondrial perturbation occurs as upstream events.

In conclusion, we report that SBL, a multifunctional protein shows cytotoxicity for some human leukemia cell lines including MDR cells (Fig. 8). The details of apoptotic signal induced by SBL was analyzed by combinational usage of specific caspase inhibitors and the mitochondrial membrane depolarization detector JC-1. The use of this combination was shown in detail to distinguish the apoptotic pathway. SBL displays novel mechanistic and tumor-selective cytotoxic effects regardless of P-gp expression and SBL has potential as an alternative molecule to conventional DNA-damaging anticancer drugs.

## Figures and Tables

**Table I.** Inhibitory effect of SBL, ETO and DOX on the viability of human leukemia cell lines.

		IC_50_ (mM)		
Cell name	Characteristics	SBL	ETO	DOX
Jurkat	T-cell leukemia	0.15±0.07	2.09±0.78	1.10±0.71
K562	Erythroleukemia	1.39±0.92	13.23±3.86	3.51±1.84
K562/ADM	P-glycoprotein-overexpressing K562 cells	0.36±0.18	N/A	N/A
U937	Promyelocytic leukemia	0.81±0.24	0.46±0.09	0.34±0.27
Raji	Burkitt’s lymphoma	0.88±0.54	0.49±0.04	0.28±0.19

Cells were treated with SBL, ETO or DOX for 72 h. IC_50_ is the concentration which resulted in a 50% decrease in cell viability. Each value indicates the mean ± SD of three different experiments performed in triplicate.
